# Institutional Questions

**Published:** 1900-07-14

**Authors:** 


### INSTITUTIONAL QUESTIONS.

**Hospital Construction: Comparison of Cost.**

"Professor" writes: May I ask you to direct me to any publications on hospital management, &c., which will give a comparison of the cost of building and equipment? We take annually "Hospitals and Charities," and in 1898, I think, there was a review of new buildings erected during the past year, but no comparison of the cost thereof. We have found "Hospitals and Asylums of the World" very useful in guiding suggestions to the architect in connection with a new hospital now about to be built in this neighbourhood, but should be glad of further information on the point I have raised.

*** In the present issue (1900) of "Hospitals and Charities" will be found a chapter dealing with the point in question. In this section will be found a list of hospitals and institutions recently built, and where it has been possible to ascertain the cost per bed of the completed building this is given in each case, so that a useful comparison of cost can be made. We do not know of any other publication in which such a comparison will be found up-to date.

